# Oxygen-Pressure-Limited Recovery of the Hematite α‑Fe_2_O_3_(0001) Surface from a Reduced Fe_3_O_4_(111)-like Layer

**DOI:** 10.1021/acs.jpcc.6c04691

**Published:** 2026-08-14

**Authors:** Nishant Kumar, Matthias Blatnik, Jan Čechal

**Affiliations:** † Brno University of Technology, 232848 CEITEC–Central European Institute of Technology, Purkyňova 123, Brno 612 00, Czech Republic; ‡ Institute of Physical Engineering, Brno University of Technology, Technická 2896/2, Brno 616 69, Czech Republic

## Abstract

The oxidation kinetics of hematite α-Fe_2_O_3_(0001) surfaces are vital for its applications in catalysis, environmental remediation, and industrial processes. Despite prior studies, the roles of temperature, oxygen partial pressure, and oxygen chemical potential in controlling nucleation and growth kinetics are not fully understood. Using real-time Low Energy Electron Microscopy/Diffraction (LEEM/LEED), we systematically investigate the oxidation of a reduced Fe_3_O_4_(111)-like surface layer to hematite under controlled conditions. We show that complete recovery of the hematite surface termination is closely linked to the nucleation and lateral growth of a two-dimensional honeycomb (H) phase. H-phase nucleation and growth do not follow standard Arrhenius kinetics. A set of experiments comprising oxidation at constant oxygen partial pressure, constant temperature, and constant chemical potential, while varying temperature, pressure, or both, indicates that oxygen supply limits the H-phase growth rate. Under constant oxygen partial pressure *p*_O2_, increasing the temperature accelerates nucleation but decelerates the growth rate of the H-phase. The growth dramatically slows for *p*_O2_ < ∼2 × 10^–6^ mbar. Keeping the oxygen chemical potential constant, i.e., regulating both *p*_O2_ and temperature to keep the thermodynamic driving force provides increasing growth rates with increasing temperature only if *p*_O2_ > ∼2 × 10^–6^ mbar. Our study thus elucidates the interplay between thermodynamics and kinetics in hematite surface oxidation, informing strategies to optimize surface properties for catalytic and industrial processes.

## Graphical abstract

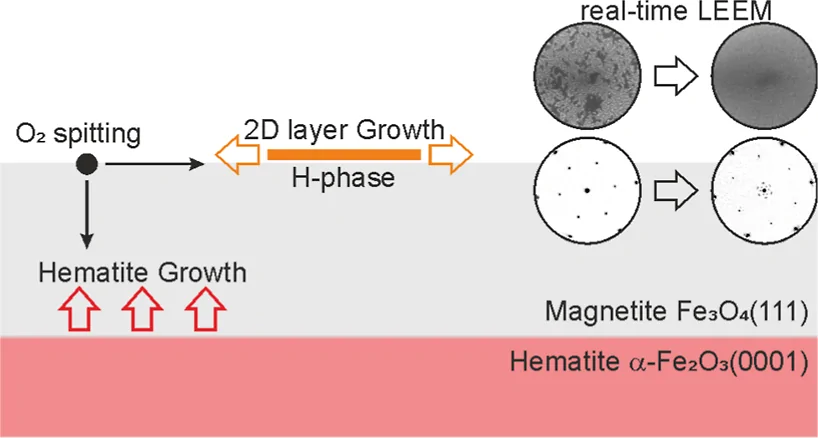

## 1 Introduction

Hematite (α-Fe_2_O_3_) is an abundant, low-toxic, catalytic, and photocatalytic material employed for CO oxidation, water purification, liquid fuel synthesis, styrene production, and water splitting.^1–3^ Because catalytic reactions occur on the surface of materials, a detailed understanding of surface structure and properties is essential for a reliable description and modeling of surface reactions. Accordingly, the hematite α-Fe_2_O_3_(0001) surface has been extensively studied using surface science techniques under ultrahigh vacuum (UHV) environment.^1^ While UHV conditions enable atomic-level insights, these studies are necessarily limited to low pressures. When the sample is treated at a low oxygen chemical potential below μ_O_ < −2.5 eV or subjected to oxygen-selective sputtering during surface preparation, hematite α-Fe_2_O_3_ is reduced,^1,4,5^ resulting in the formation of an Fe_3_O_4_(111) layer that is epitaxially matched to the underlying hematite α-Fe_2_O_3_(0001). This process creates an atomically sharp Fe_3_O_4_(111)/α-Fe_2_O_3_(0001) interface.^6^ Subsequent annealing under appropriate O_2_ partial pressures at μ_O_ > −2.5 eV restores stoichiometric α-Fe_2_O_3_(0001),^1^ which is terminated by a two-dimensional layer referred to as the honeycomb phase (H-Phase),^6,7^ a reconstructed overlayer with a characteristic moiré pattern. Within this chemical potential range, multiple bulk and surface phases coexist, complicating the preparation of well-defined samples.^1^ In addition to thermodynamic factors, the oxidation temperature and oxygen partial pressure strongly influence the oxidation kinetics; full oxidation of a reduced surface layer can require from tens of minutes to several hours, depending on conditions.^5,6,8^ Despite its importance for preparing well-defined surfaces, the kinetics of this transformation have not been quantitatively studied.

In this work, we employ low-energy electron microscopy (LEEM)^9–14^ to monitor oxidation Fe_3_O_4_(111)-like layers on α-Fe_2_O_3_(0001) in real space and time. We systematically investigate how nucleation and growth of the H-phase depend on oxygen partial pressure and temperature, revealing that the kinetics do not follow simple Arrhenius (temperature-activated) kinetics. Notably, we find that oxidation becomes slower with increasing temperature when the oxygen partial pressure is held constant, highlighting the critical role of oxygen supply.

Both hematite and magnetite are iron oxides with close-packed O^2–^ anion sublattices, but differ in their Fe cation arrangements.^1^ Hematite α-Fe_2_O_3_ has a corundum structure with Fe^3+^ in octahedral sites. Magnetite Fe_3_O_4_ has an inverse spinel structure where Fe^3+^ ions occupy tetrahedral sites, and both Fe^2+^ and Fe^3+^ ions share octahedral sites in a 1:1 ratio. Upon oxidation, Fe^2+^ is converted to Fe^3+^ within the spinel structure, leading to the formation of Fe vacancies in the octahedral sublattice; if the spinel framework is preserved during this process, the resulting phase is maghemite γ-Fe_2_O_3_ with a cation-deficient spinel structure.^1^

Magnetite to hematite transformation, known as martitization, is significant in geological sciences^15^ and magnetite ore processing.^15,16^ Two main martitization pathways are recognized: (i) an oxidizing reaction requiring molecular oxygen and (ii) a nonredox acid–base hydrothermal replacement reaction in contact with a liquid water environment via aqueous ferric species.^17,18^ In the oxidizing pathway, martitization proceeds in two-steps: formation of maghemite γ-Fe_2_O_3_ via Fe cation vacancy diffusion^19–22^ followed by the gradual transformation of maghemite γ-Fe_2_O_3_ to hematite α-Fe_2_O_3_.^1,17,23^ The conversion of metastable γ-Fe_2_O_3_ into α-Fe_2_O_3_ involves a structural rearrangement from the face-centered cubic (fcc) spinel O^2–^ sublattice to the hexagonal close-packed (hcp) corundum anion sublattice. However, under oxidizing conditions close to ours, the maghemite phase can be localized only in the vicinity of the hematite bulk, directly transforming to hematite.^24^

Further insights into magnetite oxidation have been obtained from LEEM studies on the (100) magnetite surface termination.^12,13^ Under low oxygen partial pressure (∼10^–6^ mbar) and temperatures around 650 °C, Fe cation diffusion is mediated by Fe vacancies.^21,22^ Magnetite to hematite transformation proceeds through two spatially separated reactions:^12^ at the magnetite surface O_2_ molecules dissociate and oxidize Fe^2+^ to Fe^3+^, which leads to the formation of iron vacancies in magnetite. These vacancies readily diffuse over micrometer distances within the magnetite lattice^12,19,25^ and subsequently facilitate the formation of hematite via a topotactic reaction that conserves oxygen. The ionic flow is balanced by a flow of electrons enabled by the good electrical conductivity of magnetite.^26^ In the case of Fe_3_O_4_(100) oxidation, the hematite forms inclusions inclined by 45° with respect to the surface plane, since the Fe_3_O_4_{111} planes match the α-Fe_2_O_3_(0001) plane, coinciding with the sharp interface between the Fe_3_O_4_(111) layer on α-Fe_2_O_3_(0001) bulk observed by TEM.^6^ The role of intermediate maghemite formation remains unclear. While there was no evidence for intermediate maghemite formation from electron spectroscopies,^13^ studies of 5 nm Fe_3_O_4_(111) films on Pt(111) have shown that the films initially become nonstoichiometric toward maghemite, and even after hematite forms at the surface, the underlying layers retain the spinel structure.^27^ Under oxidizing conditions, the equilibrium concentration of cation vacancies lowers with increasing temperature.^22,28^ At high temperatures (900 °C–1400 °C), this concentration increases with pressure *p*_O2_ as ${\left(\frac{p_{O_{2}}}{p^{*}}\right)}^{2/3}$ , with p* being atmospheric pressure.^22^ At lower temperatures (200 °C–400 °C), the Fe vacancy concentration is much lower than bulk equilibrium values.^19^ For Fe_3_O_4_(100) oxidation, the reaction rate significantly decreased for oxygen partial pressures below 1.3 × 10^–6^ mbar,^12^ likely due to the kinetics of vacancy formation rather than to their equilibrium concentration. In contrast to the Fe_3_O_4_(100), the oxidation of Fe_3_O_4_(111) results in α-Fe_2_O_3_(0001); the [0001] direction of hematite exhibits a polar alternation of Fe and O planes, necessitating compensation for the surface polarity. This compensation can occur via impurity segregation or by the H-phase termination.^6^ Hence, two processes occur concurrently: transformation of magnetite to hematite in the bulk and growth of the surface H-phase. Full magnetite to hematite oxidation is not possible without the presence of the polarity-compensating H-phase.

By employing real-time LEEM, we monitor surface transformations with high spatial and temporal resolution, capturing both the nucleation and growth of the two-dimensional H-phase layer during oxidation. In this study, we systematically investigate how H-phase growth kinetics is affected by independently controlling oxygen partial pressure (*p*_O2_), temperature, and oxygen chemical potential. This approach allows us to disentangle the individual contributions of these parameters to H-phase nucleation and growth, thereby clarifying their roles in the overall reaction kinetics.

## 2 Methods

Sample preparation, LEEM, and XPS characterization were performed in an ultrahigh-vacuum (UHV) system at the CEITEC Nano Research Infrastructure. The system comprises multiple UHV chambers interconnected by a UHV transfer line, allowing sample transfer between chambers via the UHV transfer line, maintaining a base pressure of 2 × 10^–10^ mbar.

### 2.1 Sample Preparation

A natural α-Fe_2_O_3_(0001) single crystal was acquired from SurfaceNet GmbH. To avoid potential interference from impurities in natural crystals, synthetic hematite films were prepared by pulsed laser deposition (PLD) in a dedicated setup with in situ ultrahigh vacuum (UHV) transfer to a surface-science chamber. The film of ∼100 nm thickness was grown on the natural α-Fe_2_O_3_(0001) single crystal from an iron oxide target using a KrF excimer laser (Coherent Compex Pro 201, λ = 248 nm) at 5 Hz pulse frequency and 2.0 J cm^–2^ laser fluence. The substrate was heated from the back by a 980 nm continuous-wave infrared laser (DILAS Compact Evolution), and its temperature was monitored with an Impac IGA5 pyrometer. Deposition was performed at 850 °C substrate temperature in 2 × 10^–2^ mbar O_2_, with a heating ramp rate of 35 °C min^–1^.

Prior to film deposition, the natural sample underwent cleaning by multiple sputtering-annealing cycles: 10 min of sputtering with 5 × 10^–7^ mbar Ar^+^ at 1 keV, followed by 10 min of annealing at 600 °C in 1 × 10^–6^ mbar O_2_. This procedure was repeated until no further changes in contaminant signals were observed in the XPS analysis. Subsequently, the sample was annealed at 800 °C for 1 h under 5 × 10^–5^ mbar O_2_ to facilitate surface morphology flattening. The cleanliness was confirmed by LEED and XPS.

The sample featuring the synthetic film was used for all experiments. To reduce the as-prepared α-Fe_2_O_3_(0001) near-surface volume to the magnetite phase (Fe_3_O_4_), the sample was subjected to four cycles of Ar^+^ sputtering (10 min cycle, 9 × 10^–6^ mbar, 1 keV, 10 mA) followed by annealing in UHV at 640 °C for 10 min.

### 2.2 Low-Energy Electron Microscopy/Diffraction

Experiments were conducted using a Specs FE-LEEM P90 instrument. Bright-field images were measured by capturing electrons collected from the central (0,0) diffracted beam. Diffraction patterns were acquired from a surface area of approximately 7 × 15 μm^2^ without aperture confinement. The coverage of the H-phase was determined using ImageJ software as the difference between the areas of the R- and H-phase regions.

X-ray Photoelectron Spectroscopy (XPS) analysis was performed using a Specs system equipped with a Phoibos 150 spectrometer. Measurements were performed with a nonmonochromatized X-ray source emitting Mg Kα radiation (*h*ν = 1253.6 eV), at 0° emission angle, utilizing the high-magnification mode with a 15 mm iris aperture. Core-level spectra were acquired with a pass energy of 20 eV, a step size of 0.1 eV, and a dwell time of 0.1 s. No charging effects were observed during the measurements.

## 3 Results

### 3.1 Initial Surface Preparation and Characterization

To establish a stable, controlled starting surface for the oxidation studies, we used an α-Fe_2_O_3_(0001) hematite single crystal featuring the 2D honeycomb phase fully covering the surface. This surface is referred to as the H-phase in the following text. Samples featuring H-phase were subsequently reduced to magnetite Fe_3_O_4_(111) through four cycles of Ar^+^ sputtering and UHV annealing at 640 °C (see Methods section for details). Sputtering selectively removes surface oxygen atoms, reducing the near-surface volume to stoichiometric Fe_3_O_4_, forming a ∼20–25 nm thick layer that maintains the epitaxial relationship with the bulk Fe_2_O_3_ hematite.^6^ We will refer to this surface as the R-phase in the following text.

The transition between the H- and R-phases was characterized using X-ray Photoelectron Spectroscopy (XPS) and Low Energy Electron Microscopy/Diffraction (LEEM/LEED). In the XPS spectrum of the R-phase (Figure 1a), the Fe^3+^ satellite peaks are absent, whereas in the H-phase spectrum (Figure 1b), the satellite peaks are present, confirming the oxidation state.^29–31^ LEEM/LEED provide complementary real and reciprocal-space views, elucidating the sample morphology at the mesoscale and its structure, respectively. Bright-field LEEM imaging revealed a uniform and well-prepared R-phase surface (Figure 1c).^7^ The corresponding diffraction pattern in Figure 1d displays (2 × 2)-Fe_3_O_4_(111) diffraction spots (referred to the O planes^6^) characteristic of this surface.^6,7,32^ The oxidation of the surface at temperatures of 660–700 °C and oxygen partial pressures of 6.3 × 10^–7^–5.0 × 10^–6^ mbar leads to full oxidation and to α-Fe_2_O_3_(0001) featuring the H-phase; depending on the oxidation conditions, it takes 55 to 450 min. The bright-field LEEM image of the H-phase (Figure 1e) shows a uniform surface, and the diffraction pattern (Figure 1f) exhibits the characteristic flowered pattern^30,32,33^ around the bulk spots associated with the moiré due to the presence of the overlayer.^7^ We note that the maghemite surface can exhibit the same (2 × 2) surface reconstruction as magnetite;^34^ therefore, maghemite and magnetite cannot be distinguished by simple diffraction experiments alone.

**Figure 1:**
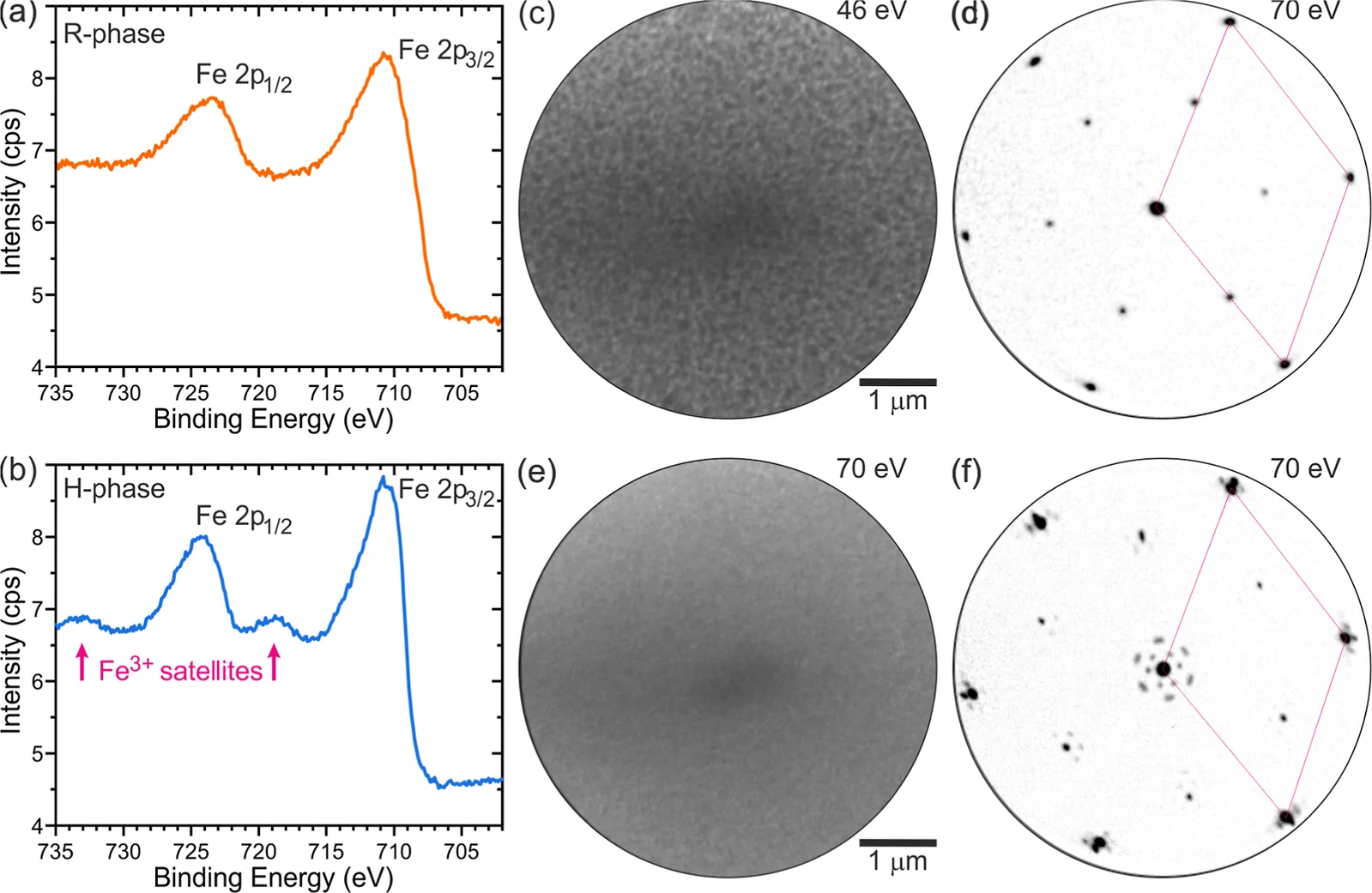
Surface characterization of hematite (H-phase) and reduced magnetite (R-phase). (a) XPS spectrum of the R-phase, confirming the absence of Fe^3+^ satellite peaks and the successful reduction of α-Fe_2_O_3_ to Fe_3_O_4_. (b) XPS spectrum of the H-phase, highlighting the presence of Fe^3+^ satellite peaks characteristic of hematite. (c) Bright-field LEEM image at 46 eV of the magnetite (R-phase) surface, showing a uniform and well-prepared morphology. (d) (2 × 2) diffraction pattern at 70 eV characteristic for the R-phase, indicative of the well-ordered surface suitable for oxidation studies. (e) Bright-field LEEM image at 70 eV showing the H-phase surface. (f) Diffraction pattern at 70 eV characteristic of the H-phase. The (1 × 1) unit cell is marked by a rhombus in (d,f).

### 3.2 Time Evolution of Oxidation

To obtain quantitative insights into the R- to H-phase transformation, we performed three sets of real-time LEEM/LEED experiments, keeping constant either oxygen partial pressure *p*_O2_, sample temperature *T*, or oxygen chemical potential μ, while varying either temperature or pressure, or in the last case, both parameters. By systematically separating the effects of these parameters, we revealed their impact on the R- to H-phase oxidation kinetics. An example of the oxidation kinetics at 660 °C and 6.3 × 10^–7^ mbar (μ_O_ = −1.867 eV) using real-time LEEM/LEED is given in Figure 2. During the first stage of oxidation, the entire surface shows a uniform contrast and the diffraction pattern of the R-phase. At 75 min, moiré spots became visible, which indicates H-phase nucleation (Figure 2a). We refer to this time, *t*_0_, as the nucleation time in the following. As oxidation progresses, the H-phase spots become more pronounced, while the R-phase spots progressively fade.

**Figure 2:**
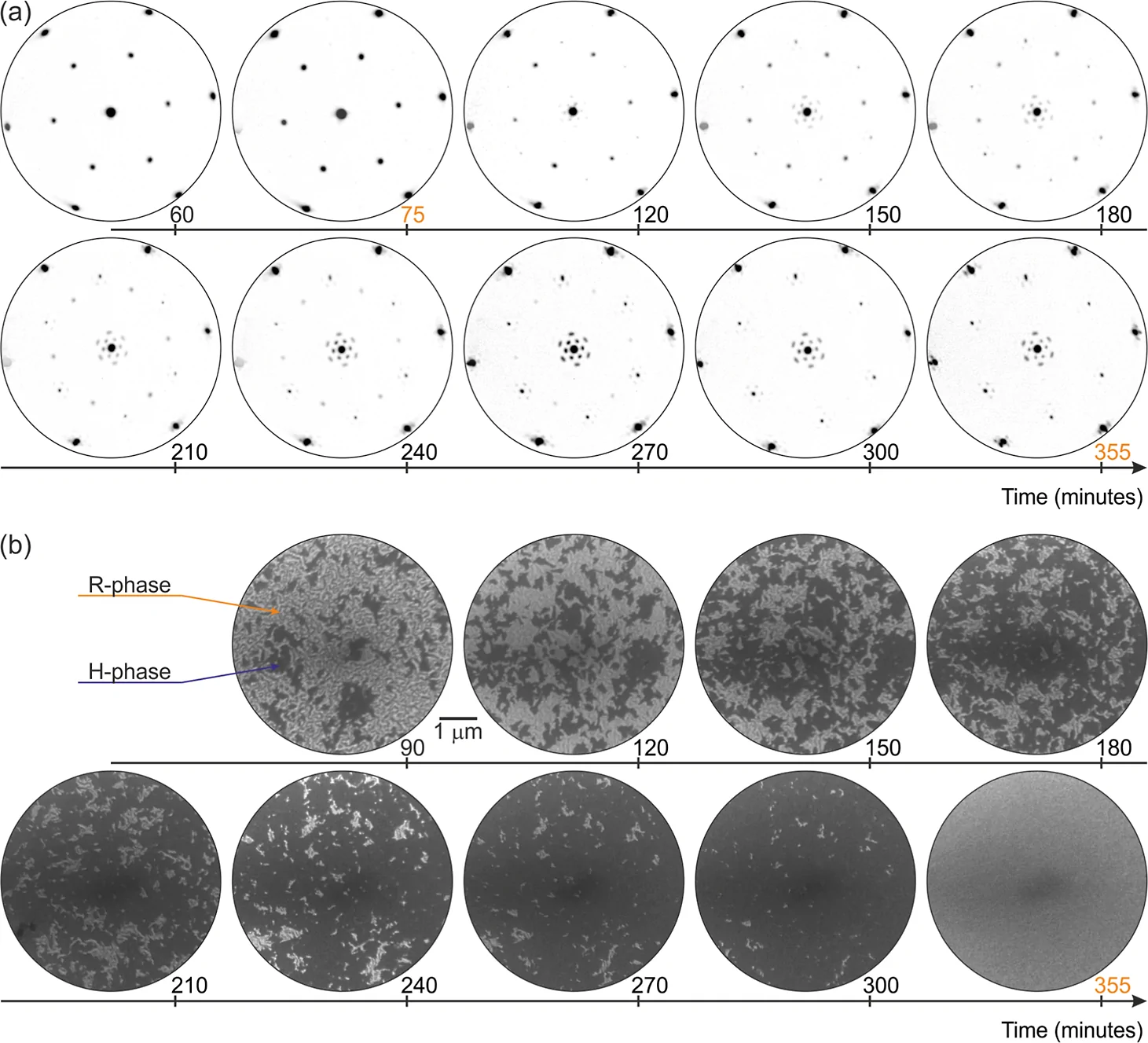
Surface evolution during the oxidation from magnetite to hematite at 660 °C and 6.3 × 10^–7^ mbar in real and reciprocal space. (a) Sequence of diffraction patterns taken at 70 eV showing the structural transformation from the R-phase to the H-phase. (b) Corresponding sequence of bright-field LEEM images capturing the transformation from the R-phase (brighter) to the oxidized H-phase (darker) over 355 min.

The bright-field LEEM image in Figure 2b illustrates the progressive transformation of the surface from the reduced R-phase to the H-phase. After nucleation, H-phase islands emerge and remain surrounded by the R-phase. As the oxidation advances, the R-phase regions on the surface gradually decrease, while the H-phase regions expand. By 355 min, the entire surface is covered by the H-phase, marking the completion of the oxidation process. The growth of the H-phase proceeds in a granular fashion, consistent with the rough growth front observed during oxidation of thin films.^24^ The evolution of H-phase coverage, derived from a series of real-space LEEM images, is shown in Figure 3. In this figure, we also define all characteristic times. Nucleation time *t*_0_ is marked by the green dashed line. Time *t*_100_, defined as the time from the start of oxidation to complete transformation, is referred to as the total time to full oxidation; we will use the derived parameter *t*_100_–*t*_0_, which gives the H-phase growth time. These parameters are shown in Figure 4 for experiments in which the pressure, temperature, or chemical potential was kept constant. The complementary reciprocal quantities, i.e., inverse nucleation time and growth rate, are provided in Figure S1 in Supporting Information. A supplementary parameter, the characteristic growth time *t*_50_, was obtained as the inverse of the growth rate determined from the slope of a linear fit of the coverage–time dependence around 50% H-phase surface coverage. The characteristic growth time yields results consistent with the H-phase growth time, as shown in Figure S2 in Supporting Information. All the values are summarized in Table S1.

**Figure 3:**
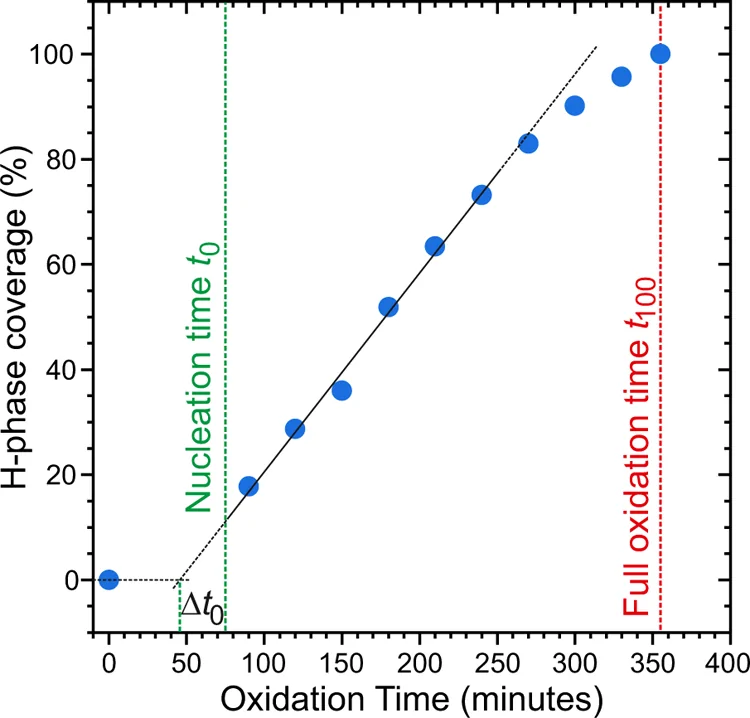
Coverage vs time graph quantifying the H-phase surface coverage (%) from the sequence of the bright-field LEEM images during oxidation from magnetite to hematite at 660 °C and 6.3 × 10^–7^ mbar (μ_O_ = −1.867 eV). The marked nucleation time is the first occurrence of the H-phase-related diffraction spots. The full oxidation marks the full substrate covered in the bright-field LEEM. The error in nucleation time was defined as the difference between *t*_0_ and the time extrapolated from the linear increase to zero coverage.

**Figure 4:**
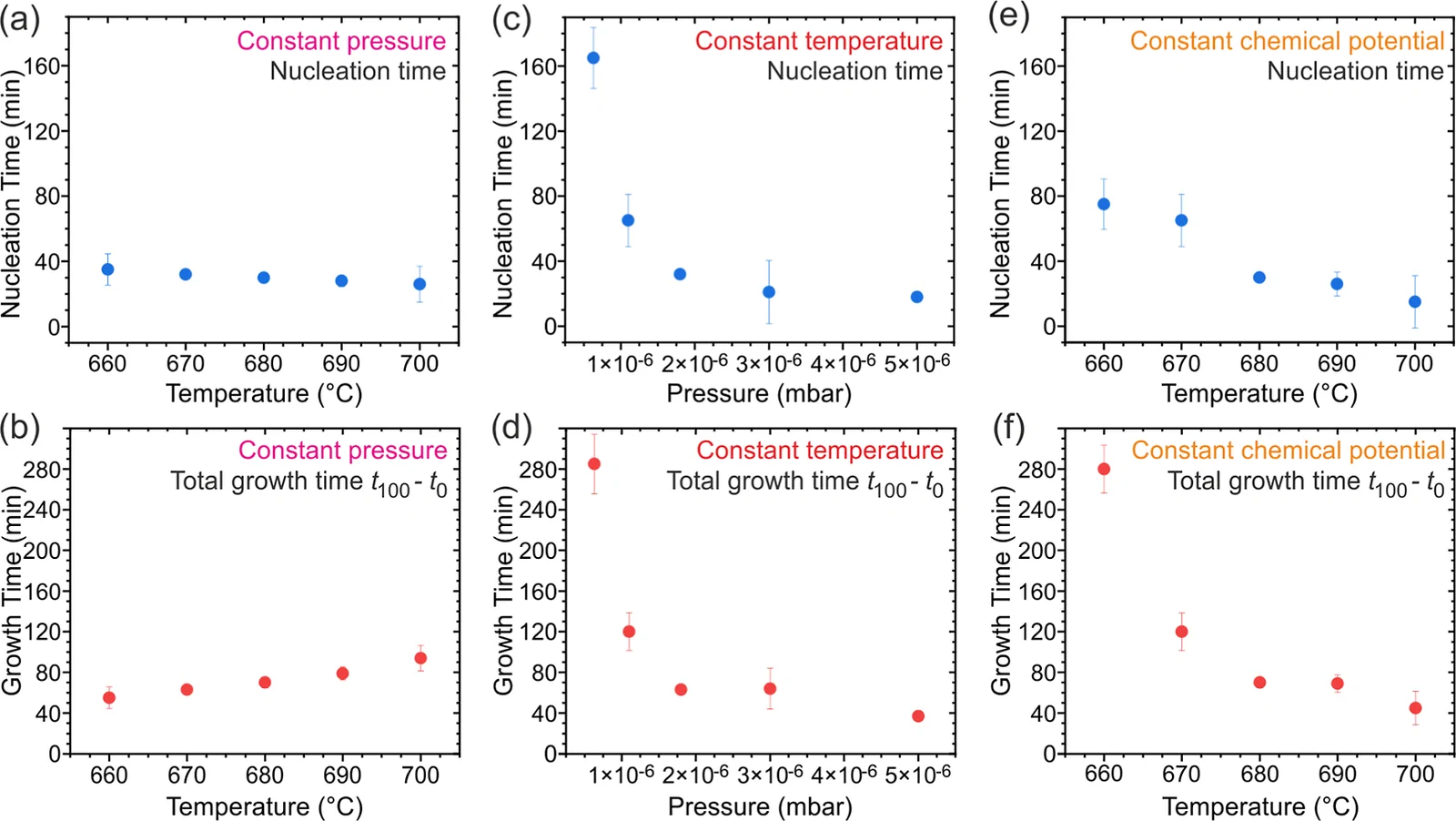
Characteristic R-phase to H-phase transformation times under varying experimental conditions. (a,b) Nucleation time (*t*_0_) and total growth time (*t*_100_–*t*_0_) as functions of temperature at constant *p*_O2_ = 1.8 × 10^–6^mbar. (c,d) Nucleation time (*t*_0_) and total growth time (*t*_100_–*t*_0_) as functions of *p*_O2_ at a constant temperature of 670 °C. (e,f) Nucleation time (*t*_0_) and total growth time (*t*_100_–*t*_0_) as functions of temperature under constant oxygen chemical potential μ_O_ = −1.867 eV. Error bars represent one standard deviation, reflecting the uncertainty of deriving the given parameter; if the error bars are small compared to the symbol size, they are not displayed.

### 3.3 Oxidation at Constant Oxygen Partial Pressure

We applied the real-time real-space LEEM/LEED methodology described above to study the oxidation kinetics under a range of temperatures and pressures. We start with the standard experiment in which we have varied the temperature and kept the oxygen partial pressure constant (*p*_O2_ = 1.8 × 10^–6^ mbar). The characteristic time *t*_0_ for the onset of nucleation, and the total growth time *t*_100_–*t*_0_, as a function of temperature, are displayed in Figure 4a,b.

Nucleation time *t*_0_ slightly decreased with increasing temperature. At 660 °C, nucleation required 35 min, whereas at 700 °C, it decreased to 26 min. This behavior aligns with thermally activated processes, in which higher temperatures increase the rate of the rate-limiting step. Conversely, the growth time *t*_100_–*t*_0_ increased with temperature. The total growth time *t*_100_–*t*_0_ increases approximately linearly with temperature; at 660 °C, the H-phase growth time was 55 min, whereas it extended to 94 min at 700 °C (Figure 4b). The associated inverse nucleation time shows approximately linear increase with temperature, whereas the growth rate decreases with temperature (Figure S1 in Supporting Information).

### 3.4 Oxidation at Constant Temperature

We observe that increasing temperature alone does not result in an increased oxidation rate. To further investigate this, we examined the oxidation kinetics under a constant temperature of 670 °C while systematically varying the *p*_O2_. Changes in oxygen pressure influence both nucleation and growth. Both nucleation time *t*_0_ and growth time *t*_100_–*t*_0_ decreased with increasing *p*_O2_ as shown in Figure 4c,d. At the lowest pressure of 6.3 × 10^–7^ mbar, nucleation required approximately 165 min, decreasing significantly to 18 min at the highest pressure of 5.0 × 10^–6^ mbar. Similarly, the H-phase growth time *t*_100_–*t*_0_ (Figure 4d) decreases from 285 to 37 min with increasing pressure. There is a dramatic increase in both times in the low-pressure range, most clearly below ∼2.0 × 10^–6^ mbar. Considering the reciprocal times (the inverse nucleation time and the growth rate), these increase with pressure in the low-pressure regime and tend to saturate at higher pressures (Figure S1 in Supporting Information). Thus, low oxygen pressure significantly limits the oxidation rate, while further increases above a certain threshold have a diminishing effect.

### 3.5 Oxidation at Constant Oxygen Chemical Potential

Informed by the fact that oxygen availability is a critical factor, we performed a temperature-dependent study, but under a constant oxygen chemical potential, by simultaneously varying pressure to keep the oxygen chemical potential constant at μ_O_ = −1.867 eV according to^35^ μ_O_ = 1/2 [μ_O2_^0^(*T*) + *kTln*(*p*_O2_/*p* ^0^)], where *k* is the Boltzmann constant, and *T* is the absolute temperature in Kelvin, *p*_O2_ is the oxygen partial pressure, and *p* ^0^ = 1 bar. The reference chemical potential μ_O2_^0^(*T*) = *H*_O2_(*T*,*p* ^0^) – *H*_O2_(0,*p* ^0^) – *T*[*S*_O2_(*T*,*p* ^0^) – *S*_O2_(0,*p* ^0^)] is chosen so that μ_O2_(0,*p*_O2_) = 0;^36^ the enthalpy *H*_O2_(*T*,*p* ^0^) and entropy *S*_O2_(*T*,*p* ^0^) per O_2_ molecule are taken from thermochemical tables.^37^

At constant chemical potential, the nucleation times *t*_0_ exhibited a stronger temperature dependence than at constant *p*_O2_. It decreased from 75 to 15 min when the temperature increased from 660 to 700 °C (Figure 4e). The constant-pressure study was performed at an intermediate pressure of 1.8 × 10^–6^ mbar with the chosen chemical potential corresponding to this pressure at 680 °C. To keep the chemical potential constant, lowering the temperature must be accompanied by a decrease in pressure, which prolongs nucleation time. Conversely, at higher temperatures, the pressure is higher, and the nucleation time is decreased. The H-phase growth time *t*_100_–*t*_0_ (Figure 4f) first decreases from 280 to 70 min, followed by a smaller decrease to 45 min beyond 680 °C. These findings emphasize the strong role of oxygen partial pressure and oxygen supply, even when the thermodynamic driving force is kept constant.

## 4 Discussion

The initial state is a layer of magnetite on bulk hematite. Hence, during the oxidation, the reduced layer should be reoxidized, and the overlayer termination formed (H-Phase). BF-LEEM imaging reveals that H-Phase islands nucleate at multiple locations and grow laterally. Throughout oxidation, the H-phase islands remain surrounded by R-phase, which only disappears once the surface is fully covered by H-phase. As the [0001] direction of hematite exhibits a polar alternation of Fe and O planes, the H-phase termination provides the necessary polarity compensation.^6^ Across all experimental conditions, we consistently observe that complete recovery of the hematite surface termination is closely associated with the presence of the H-phase layer.

Our data also show that the growth does not follow simple thermally activated (Arrhenius) kinetics, as the supply of oxygen for the reaction is a critical factor. When the temperature is increased at constant oxygen partial pressure, the nucleation rate increases but the growth rate decreases, indicating that nucleation and growth are governed by distinct kinetic pathways. The observed decrease in the growth rate suggests that the reaction is not solely controlled by thermal activation, but is also limited by oxygen availability. To enhance the growth rate, the increase in temperature should be accompanied by a corresponding increase in oxygen partial pressure. On the other hand, the growth rate decreases dramatically at oxygen partial pressures below 2 × 10^–6^ mbar. In this pressure range, the growth rate increases with pressure, while further increase above 2 × 10^–6^ mbar has a diminishing effect on the kinetics. Under these low-pressure conditions, maintaining constant chemical potential by lowering the temperature does not compensate for the limiting effect of low pressure, as the pressure dependence dominates. A similar decrease for oxygen partial pressures below 1.3 × 10^–6^ mbar was also observed in the case of Fe_3_O_4_(100) oxidation.^12^

Considering the oxidation mechanism, the key step is the formation of Fe vacancies via oxygen dissociation at the magnetite surface, followed by their subsequent transport.^12^ Previous studies on the Fe_3_O_4_(100) have shown that the oxidation rate is limited by the kinetics of Fe vacancy formation rather than their equilibrium concentration. The observed increase in growth time with increasing temperature thus suggests a competing process, which could be either O_2_ desorption during oxygen splitting or the diffusion of Fe vacancies into the bulk. As shown in Figure 3, the growth rate remains uniform until the final stages of oxidation. Given that dissociative oxygen adsorption occurs uniformly across the magnetite surface,^12^ significant local variations in growth rate would be expected if the supply of dissociated oxygen from terraces were the sole limiting factor. This observation indicates that the transport of reaction-mediating defects, such as Fe vacancies, to the reaction site is a critical factor in determining the overall oxidation rate.

Our results suggest a more complex nature of H-phase growth, involving two distinct oxidations: oxidation of bulk magnetite and formation of the terminating H-phase layer, with bulk oxidation not appearing to be completed at the surface in the absence of the H-phase. Elucidating the relationship between these two oxidation processes would benefit from a combined approach using LEEM and cross-sectional TEM imaging,^6^ which we plan to pursue in future work.

## 5 Conclusions

In conclusion, real-time LEEM/LEED shows that oxidation of the reduced Fe_3_O_4_(111)-like surface layer on α-Fe_2_O_3_(0001) is accompanied by nucleation and lateral growth of the H-phase. The growth kinetics show a significant dependence on oxygen supply for the surface reaction. While a slight increase in temperature slightly accelerates H-phase nucleation, lateral growth slows at constant oxygen pressure, indicating that the supply of reactive oxygen limits the surface reaction. Increasing temperature must therefore be accompanied by a sufficient increase in oxygen partial pressure to sustain the oxidation rate. Below an oxygen partial pressure of ∼2 × 10^–6^ mbar, the H-phase growth rate decreases dramatically, and this decrease cannot be compensated simply by lowering the temperature. The observation that residual R-phase persists until H-phase coalescence shows that complete recovery of the hematite surface termination is closely coupled to lateral H-phase growth. These results indicate a general strategy for faster oxidation of the reduced Fe_3_O_4_(111)-like surface layer, in which the pressure should be above a certain threshold and the increase in temperature should be accompanied by an increase in pressure to keep the thermodynamic driving force constant. This knowledge is important for fundamental studies of this catalytically relevant surface.

## Supplementary Material



## Data Availability

The data that support the findings of this study are available from the corresponding author upon reasonable request.

## Notes

Supporting Information a table summarizing the experimental data and figures showing the nucleation/oxidation rates and dependence of parameter *t*_50_ (PDF)
