# Supplementary material for: Oxygen-Pressure-Limited Recovery of the Hematite α‑Fe2O3(0001) Surface from a Reduced Fe3O4(111)-like Layer
Source: J Phys Chem C Nanomater Interfaces. 2026 Aug 14;130(34):12224–31. doi: 10.1021/acs.jpcc.6c04691 (PMC13528579; doi:10.1021/acs.jpcc.6c04691)
Supplement: Supplementary file 1 [file jp6c04691_si_001.pdf]

SUPPORTING INFORMATION

Oxygen-Pressure-Limited Recovery of the Hematite α-Fe_2O_3(0001) Surface from a Reduced Fe_3O_4(111)-Like Layer

*Nishant Kumar,[1] Matthias Blatnik,[1] Jan Čechal[1,2]**

[1] CEITEC - Central European Institute of Technology, Brno University of Technology, Purkyňova 123, 612 00 Brno, Czech Republic.

[2] Institute of Physical Engineering, Brno University of Technology, Technická 2896/2, 616 69 Brno, Czech Republic.

Table S1: Oxidation kinetics data for hematite (α-Fe_2O_3) under different experimental conditions.

	Temperature (°C)	Pressure (mbar)	Nucleation Time (t_0) [min]*	Error in t_0 (Δt_0) [min]	Characteristic phase growth time (t_{50}) [min]	Error in t_{50} (Δt_{50}) [min]	Time to full oxidation (t_{100}) [min]
Constant Partial Oxygen Pressure	660	1.8×10^{-6}	35	9.67	13.6	12.57	90
	670	1.8×10^{-6}	32	0.81	4.03	1.0	95
	680	1.8×10^{-6}	30	1.81	8.49	2.26	100
	690	1.8×10^{-6}	28	3.39	23.44	4.99	107
	700	1.8×10^{-6}	26	10.93	40.54	17.33	120
Constant Temperature	670	6.3×10^{-7}	165	18.63	104.65	26.7	450
	670	1.1×10^{-6}	65	16.02	43.18	23.45	185
	670	1.8×10^{-6}	32	0.81	4.03	1.0	95
	670	3.0×10^{-6}	21	19.5	30.21	26.29	85
	670	5.0×10^{-6}	18	1.78	10.14	2.49	55
Constant Oxygen Chemical Potential ($\mu = -1.867$ eV)	660	6.3×10^{-7}	75	15.49	104.86	24.18	355
	670	1.1×10^{-6}	65	16.02	43.18	23.45	185
	680	1.8×10^{-6}	30	1.81	8.49	2.26	100
	690	3.0×10^{-6}	26	7.33	15.04	8.48	95
	700	5.0×10^{-6}	15	16.09	16.61	19.27	60

*Nucleation times (t_0) are experimentally measured, while errors (Δt_0) are derived from the intersection of linear fit of the following linear increase with the baseline. Error for Time to full oxidation (t_{100}) is estimated to be below 5 % of its nominal value.

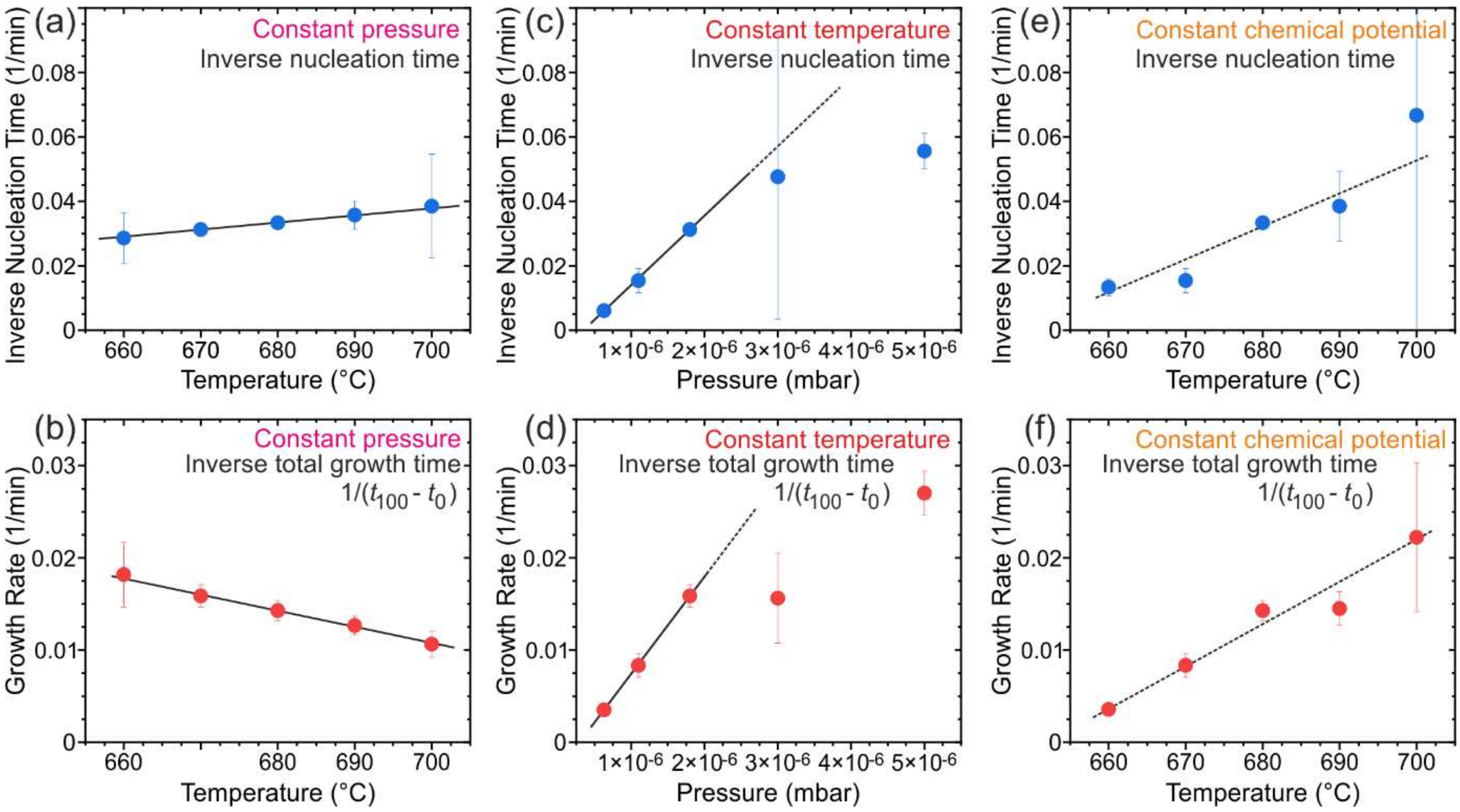

Figure S1: Inverse times associated with Figure 4 in the main text. (a, b) Inverse nucleation time (t_0) and inverse growth time t_{100}- t_0 (growth rate) as function of temperature at constant $p_{O_2} = 1.8 \times 10^{-6}$ mbar. (c, d Inverse nucleation time (t_0) and inverse growth time t_{100}- t_0 (growth rate) as function of p_{O_2} at constant temperature (670°C). (e, f) Inverse nucleation time (t_0) and inverse growth time t_{100}- t_0 (growth rate) as function of temperature under constant oxygen chemical potential ($\mu_O = -1.867$ eV).

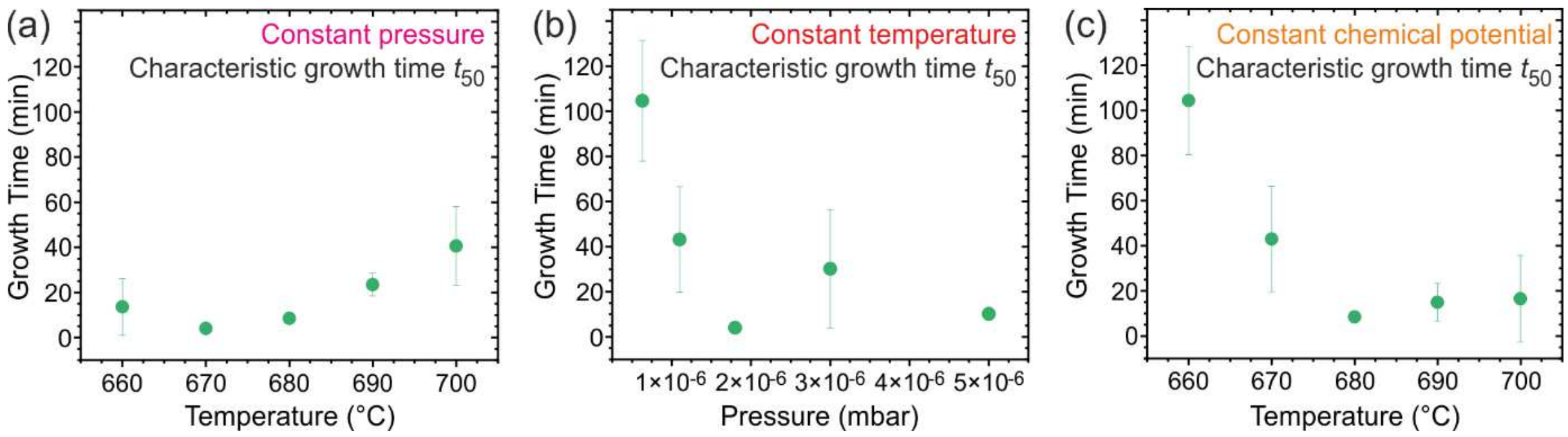

Figure S2: Characteristic H-phase growth time (t_{50}) under (a) as function of temperature at constant $p_{O_2} = 1.8 \times 10^{-6}$ mbar, (b) as function of p_{O_2} at constant temperature (670°C), and (c) as function of temperature under constant oxygen chemical potential ($\mu_O = -1.867$ eV).
